# Triplet-Based Codon Organization Optimizes the Impact of Synonymous Mutation on Nucleic Acid Molecular Dynamics

**DOI:** 10.1007/s00239-018-9828-x

**Published:** 2018-01-17

**Authors:** Gregory A. Babbitt, Erin E. Coppola, Jamie S. Mortensen, Patrick X. Ekeren, Cosmo Viola, Dallan Goldblatt, André O. Hudson

**Affiliations:** 1 T.H. Gosnell School of Life Sciences, Rochester Institute of Technology, Rochester, NY USA; 2 Biomedical Engineering, Rochester Institute of Technology, Rochester, NY USA; 3 Mechanical Engineering, Rochester Institute of Technology, Rochester, NY USA; 4 McQuaid Jesuit High School Computer Club, Rochester, NY USA; 5 Applied Mathematics, Cornell University, Ithaca, NY USA

**Keywords:** Dual-use codon, RNA world, Molecular dynamics, Codon organization, Genetic code

## Abstract

Since the elucidation of the genetic code almost 50 years ago, many nonrandom aspects of its codon organization remain only partly resolved. Here, we investigate the recent hypothesis of ‘dual-use’ codons which proposes that in addition to allowing adjustment of codon optimization to tRNA abundance, the degeneracy in the triplet-based genetic code also multiplexes information regarding DNA’s helical shape and protein-binding dynamics while avoiding interference with other protein-level characteristics determined by amino acid properties. How such structural optimization of the code within eukaryotic chromatin could have arisen from an RNA world is a mystery, but would imply some preadaptation in an RNA context. We analyzed synonymous (protein-silent) and nonsynonymous (protein-altering) mutational impacts on molecular dynamics in 13823 identically degenerate alternative codon reorganizations, defined by codon transitions in 7680 GPU-accelerated molecular dynamic simulations of implicitly and explicitly solvated double-stranded aRNA and bDNA structures. When compared to all possible alternative codon assignments, the standard genetic code minimized the impact of synonymous mutations on the random atomic fluctuations and correlations of carbon backbone vector trajectories while facilitating the specific movements that contribute to DNA polymer flexibility. This trend was notably stronger in the context of RNA supporting the idea that dual-use codon optimization and informational multiplexing in DNA resulted from the preadaptation of the RNA duplex to resist changes to thermostability. The nonrandom and divergent molecular dynamics of synonymous mutations also imply that the triplet-based code may have resulted from adaptive functional expansion enabling a primordial doublet code to multiplex gene regulatory information via the shape and charge of the minor groove.

**Electronic supplementary material:**

The online version of this article (10.1007/s00239-018-9828-x) contains supplementary material, which is available to authorized users.

## Introduction

All known life on Earth uses nearly the same genetic code to control the templated reproduction of polypeptides from nucleic acid polymers. Its triplet-based codon organization appears to serve two rather distinct functions. The most primary of these is the specification of amino acids by the correct combination of tRNA activation and codon–anticodon binding interaction guided by the first and second nucleobase. A less obvious secondary function is the accommodation of nucleic acid sequence level variation for reasons that are still largely unknown. This degeneracy in the code is achieved through the prevalence of twofold and fourfold synonymous sites which occur mostly at the third codon position and are frequently accommodated by nonspecific base pairing and tRNA wobble at the ribosome (Crick 1966). Thus, the triplet codon would appear to allow these two aspects of the genetic code to coexist without much physicochemical interference. Although it was initially suggested that the codon table was completely random in its organization and yet somehow subject to extreme purifying selection (i.e., ‘frozen accident’) (Crick 1968), it is now generally thought it was likely the product of early adaptive selection to minimize translational error (Freeland and Hurst 1998; Freeland et al. 2003) even prior to its later expansion to triplet codons (Novozhilov and Koonin 2009). Furthermore, the idea that the degeneracy in the code lends function to local genomic process is now supported by observations that local patterns of codon usage bias optimize proper levels of gene expression controlled through rates of transcription (Zhou et al. 2016), while minimizing levels of translational error and protein misfolding through local control over the rate of translation elongation (Chamary and Hurst 2005; Yu et al. 2015). Despite this functional aspect of codon usage, a rudimentary understanding of the human health consequences of sequence variation at synonymous sites still remains elusive (Bali and Bebok 2015).

It has recently been demonstrated that codon organization supports the optimization of binding interactions in eukaryotic exonic DNA (Stergachis et al. 2013). These binding interactions have a direct link to the topology of the DNA helix (i.e., DNA shape), which is largely a function of minor groove width, which in turn is defined by the relative positioning of base pairing at every third step on the DNA ladder (Parker et al. 2009; Parker and Tullius 2011). This aspect of DNA shape has been demonstrated to affect electrostatic function with respect to transcription factor binding interaction and nucleosome formation through the interaction of arginine and the minor groove (Rohs et al. 2009). Furthermore, it has also been theorized that synonymous codon organization in the genetic code might serve to accommodate the binding activity of DNA through the GC content dependence of DNA flexibility and subsequent compaction in the eukaryotic nucleus and bacterial nucleoid (Tillo and Hughes 2009; Babbitt and Schulze 2012; Babbitt et al. 2014). Thus, the convenient relation between DNA shape and the sequence variation afforded by triplet codons is perhaps no coincidence. However, it is a real mystery how such an apparently functional aspect of codons in the context of DNA could have evolved in an RNA world, where it is generally thought that the genetic code predates the evolution of the ribosome (Crick et al. 1976) as well as the use of DNA for information storage [but also see (Martin and Russell 2003)]. However, the chemical similarity of double-stranded nucleic acids might imply a correlation in molecular dynamics (MD) that could have preadapted codons to optimized patterns of use in eukaryotic DNA contexts observed in the present day. Patterns of 3 bp alternating stagger observed in the z-axis plane of the base-pairing of double-stranded RNA would also seem to impose direct relationship between RNA duplex topology and a potential 3 bp patterning in sequence variation in the RNA world that functioned similarly in protein binding to that observed by (Parker et al. 2009; Parker and Tullius 2011) regarding the shape of DNA.

Additional hints that regulatory adaptation specific to nucleic acid molecular mechanics might comprise a major organizing principle in the genetic code are implied by recent findings that (A) the code is highly optimized towards an ability to multiplex additional ‘parallel’ information related to RNA duplexing and protein-binding interaction (Itzkovitz and Alon 2007), that (B) mechanical torsion on DNA can actually control transcription rate (Ma et al. 2013), that (C) many codons can have secondary functionality or ‘dual-use’ regarding transcription factor binding in coding regions in humans (Stergachis et al. 2013), that (D) natural selection can frequently be observed acting on codon bias at synonymous sites (Chamary and Hurst 2005; Chamary et al. 2006; Hershberg and Petrov 2008, 2009; Lawrie et al. 2013), and lastly that (E) this selection is highly dependent on local sequence context (Agashe et al. 2016) perhaps affecting biophysical aspects of GC3-related polymer flexibility within synonymous protein space (Babbitt et al. 2014). Taken together, these observations imply that something of functional importance is unusually accommodated by the third base position in the genetic code, is highly correlated to simple nucleic acid biophysics through GC content, and exists relatively independent of the level of protein structure [but see (Yu et al. 2015)].

Currently, four hypotheses regarding the origin of the codon organization in the standard genetic code state that it might reflect (A) nonrandom physicochemical properties of its constituent alphabets (Crick et al. 1976; Di Giulio and Medugno 1998; Polyansky et al. 2013), (B) nonrandom co-evolutionary expansion processes prior to LUCA (Szathmáry 1993; Itzkovitz and Alon 2007; Novozhilov and Koonin 2009), (C) nonrandom adaptive evolution to minimize mutational impacts (Freeland and Hurst 1998; Freeland et al. 2003; Novozhilov and Koonin 2009), or, as originally hypothesized, (D) strong functional conservation of a nonrandom pattern that has no functional significance at all (Crick 1968). The first three hypotheses are not entirely mutually exclusive (Koonin and Novozhilov 2009), and might be unified under a more biophysically grounded analysis of the problem than typical frequentist-based bioinformatics has thus far allowed (Parker and Tullius 2011; Wilke 2012; Babbitt et al. 2016). Because most of these previous theories focus only on the primary protein coding function of the genetic code, none appropriately address the potential evolutionary expansion of the code to incorporate biophysically tuned gene regulation via synonymous codons (Babbitt et al. 2014).

To investigate this idea, we compare the relative overall impact of synonymous and nonsynonymous mutation on nucleic acid MD in an attempt to observe if they might show substantial biophysical differences in the standard genetic code that are unlikely under all possible random coding schemes. Our general hypothesis is that if synonymous sites serve to maintain the structural shape and dynamic properties of nucleic acid, then mutational impacts on the MD on these sites should differ quantitatively on the whole when compared to nonsynonymous sites. Furthermore, this dichotomy should be decidedly nonrandom when the synonymous codon organization of the real canonical code is compared to all possible random reassignments of codons in alternative codes. We employ several methods of GPU-accelerated MD simulation on both DNA and double-stranded RNA structures (Pérez et al. 2012; Foloppe et al. 2013) as well as sequence-based empirical metrics of DNA flexibility (Heddi et al. 2010) to quantify the relative total impact of nonsynonymous and synonymous mutations under all possible equally degenerate alternative code schemes. We use the distribution of these sum totals of mutational impacts under alternative codes to assess the level of nonrandom functional optimization in the genetic code regarding nucleic acid polymer dynamics. We report a fundamental pattern in the way the genetic code reduces biophysical impacts on MD by means of its synonymous codon organization. We propose that the third-base position dependent degeneracy in the genetic code might represent a functionally adaptive accessorization event that expanded a simpler doublet-based code in a way that could maintain functional biophysical constraints on nucleic acid polymer dynamics while not interfering with the protein world.

## Materials and Methods

### Overview of the Computer Simulation

**Fig. 1:**
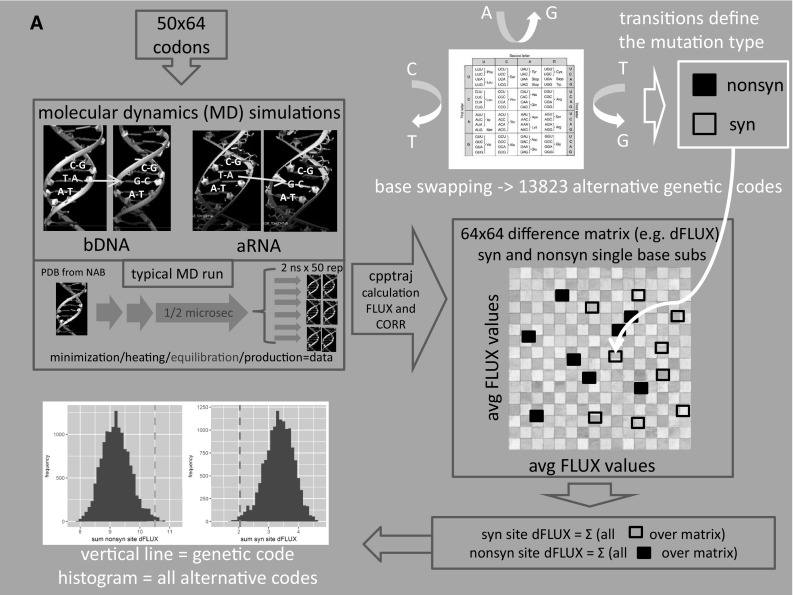

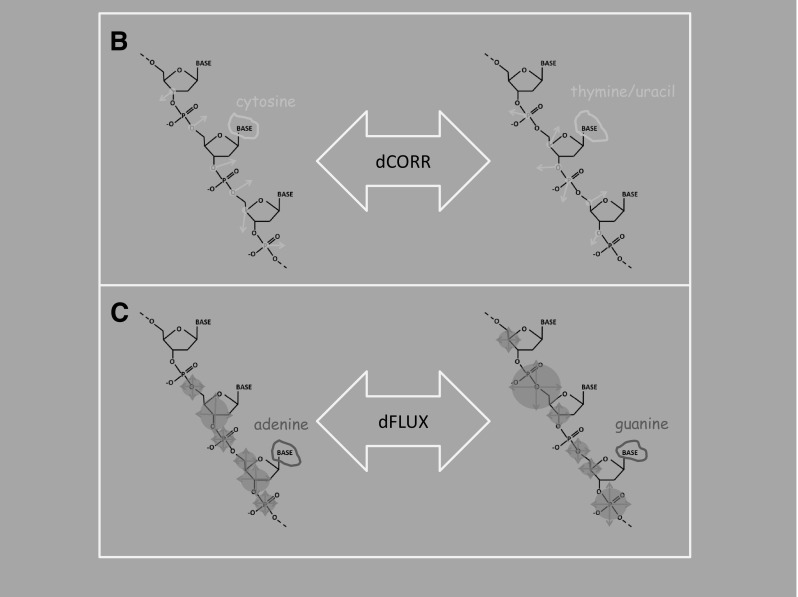
A general overview (**a**) of our pipeline for calculating the level of MD optimization within the context of nucleic acid structures (i.e., dsRNA and bDNA). Generalized Born MD simulations (Amber 14) were run on 50 sets of all 64 codons centered on implicitly solvated G-capped 23 bp DNA fragments. Explicitly solvated systems were tested as well with equilibration times reduced to 0.2 µs. Alternative coding schemes defining the synonymous/nonsynonymous categorizations were generated by combinatoric base exchanges at all three codon positions, thus maintaining the same level of degeneracy in all alternative code combinations. Mutational impacts for all changes within the genetic code were defined using atomic fluctuations and correlations (i.e., cpptraj) and then summed over all synonymous sites (blue line) and all nonsynonymous sites (yellow line) and then compared to histograms of respective synonymous or nonsynonymous impacts in all the alternative codes (histograms). Mutational impacts were defined two ways, first as (**b**) absolute differences in correlation of vector trajectories (i.e., yellow arrows) along backbone carbons, and second as (**c**) absolute differences in atomic fluctuations or rapid harmonic vibrations (i.e., blue circles). (Color figure online)

Our computational pipeline consists of five steps visually outlined in Fig. 1a. First, after minimization of the potential energy surface, subsequent heating, and a microsecond scale equilibration period, we ran 3200 subsequent replications of nanosecond scale GPU-accelerated MD simulations on both implicitly and explicitly solvated bDNA and double-stranded aRNA structures (i.e., 50 implicit sets and 10 explicit sets of all 64 codons in both molecular contexts = 7680 total simulations). Over the replicate simulations for each codon, we quantified both the average atomic correlation (CORR, Fig. 1b) and the average atomic fluctuation (FLUX, Fig. 1c) on the carbon backbone. Secondly, we defined a codon-based transition matrix quantifying the average absolute mutational impact of single-base substitutions on the MD of backbone carbons surrounding the sites of mutation in both RNA and DNA context (i.e., dCORR and dFLUX). Third, we use the degenerate codon organization of the canonical genetic code to define, as single sums, the relative total impact of synonymous and nonsynonymous mutation on these MD features of the simulated nucleic acid polymers. Fourth, we then swap base assignments randomly at each codon position to generate all possible alternative genetic codes with identical levels of degeneracy for each amino acid, while repeatedly calculating the overall synonymous and nonsynonymous mutational impacts on RNA and DNA MD for each alternate code. Lastly, we use the overall distribution of synonymous and nonsynonymous MD impacts under alternative codes to derive the empirical probability (i.e., *p* value) of the relative synonymous and nonsynonymous impacts calculated under the real genetic code (in step 3).

### Molecular Dynamic Simulation Protocols

All MD simulations were carried out sequentially on 2 Ubuntu 16.04 linux servers (Dell T430 Intel Xeon) mounting a 2880 core water-cooled 980ti and a 3072 core Titan-X GPU running pmemd.cuda released with Amber16 (Götz et al. 2012; Roe and Cheatham 2013; Salomon-Ferrer et al. 2013) and automated by our own modularized Perl/R pipeline. Our implicitly solvated simulation protocol was based on recently a published study of B-DNA under a Generalized Born model (Nguyen et al. 2015). Explicitly solvated simulations using the method of particle mesh Ewald (PME) were also conducted. PDB files for the bDNA and aRNA structures were created in the Nucleic Acid Builder program of AmberTools16 (Arnott et al. 1973, 1980). Each sequence consisted of a given codon sequence flanked with two randomly generated 11 bp sequences consisting of equal base composition. Topology and coordinate files were then generated in LeAP. Each DNA structure was loaded with the force field ff99 modified with the parmbsc0 α/γ dihedral, ε/ζ OL1 and χ OL4 parameters (OL15) (Krepl et al. 2012; Zgarbová et al. 2013). RNA structures were loaded with the force field ff99 modified with parmbsc0 α/γ dihedral and χOL3 parameters (OL3) (Pérez et al. 2007; Zgarbová et al. 2011). The salt concentration was set to 100 mM for all simulations. Each structure underwent initial energy minimization for 500 ps with a cut-off of 12 Å. The structures were then heated in four stages from 100 to 300 K under Langevin dynamics (250 ps per stage). Weight restraints were applied to all heavy atoms using a force constant of 10.0 kcal/mol-Å. The structures were then maintained at 300 K while the force constant was lowered every 250 ps (10.0, 1.0, 0.1 kcal/mol-Å) during three subsequent stages prior to the production MD runs. This was followed by a very long equilibration period run at constant temperature and pressure for 0.5 µs (0.2 µs for the explicit solvated runs).

Production MD was performed under the Hawkins, Cramer, Truhlar pairwise Generalized Born model (HCT-GB) for implicit solvation (Hawkins et al. 1996). A total of 50 × 64 implicit solvent simulations were conducted for both RNA and DNA (total of 3200 simulations each) with each of the 50 replicate runs starting with the coordinates and vector trajectories obtained at the end of the equilibration run for a given codon. Each MD simulation production run lasted for 2.0 ns (0.5E9 steps) with an integration time step of 2.0 fs and an infinite cut-off. The first 0.5 ns of each run was treated as a period of extended equilibrium. A Langevin dynamics thermostat was used to control for our constant temperature simulations (300 K) at a collision frequency of 10 ps^−1^. Bond length constraints were applied to all hydrogens using the SHAKE algorithm. Center-of-mass motion was removed every 1000 steps and slow varying forces were evaluated every 2 steps. The minimum distance calculated for the effective Born radii was 25.0 Å. Simulation data were output every 500 steps to a binary NetCDF trajectory for analysis. The protocols for explicitly solvated DNA and RNA structures were identical to those above, except that Generalized Born parameters were deactivated; structures were charged neutralized with Na^+^ in the TIP3BOX 10.0 water box; only ten replicates per codon were produced; the production run time per replicate was 0.5 ns, and the equilibration time steps for each codon were reduced to 0.2 µs.

Post-simulation trajectory and coordinate analyses for all MD runs were conducted on the Linux terminal with cpptraj and R Graphics module ggplot2 controlled with our own Perl/R pipeline. Several positional metrics were calculated in cpptraj for each run. These metrics include the 6 bp parameters (twist, roll, tilt, rise, shift, slide), root-mean-square deviation (RMSD), root-mean-square fluctuations (RMSF or ‘FLUX’), and atomic correlation (AC or ‘CORR’) averaged over time. Base pair parameters, RMSD, RMSF, and AC values per position were averaged over each data set. The calculations used all backbone atoms within a 5-mer mask centered on each simulation of 25 bp sequence. Mutational impact scores in our MD simulations were based on atomic fluctuations (i.e., simulated crystallographic B factors) and atomic correlations among single atom vector trajectories calculated along a single strand of the backbone using the 5-mer mask centered on each codon type (i.e., including the three bases in each codon plus 1 flanking base). Mutational impacts (i.e., atomic fluctuation or correlation shifts, Eqs. 1 and 2) were determined by calculating the absolute difference in the mask values before and after mutation summed over all respective synonymous or nonsynonymous single-base substitutions (i) that are possible within a given code.

1 $${\text{dFLUX}}\left( {\AA} \right)=\mathop \sum \limits_{{i=1}}^{{N~or~S}} \left| {{\text{FLU}}{{\text{X}}^{{\text{before}}}} - {\text{FLU}}{{\text{X}}^{{\text{after}}}}} \right|$$

2 $${\text{dCORR}}(r)=\mathop \sum \limits_{{i=1}}^{{N~or~S}} \left| {{\text{COR}}{{\text{R}}^{{\text{before}}}} - {\text{COR}}{{\text{R}}^{{\text{after}}}}} \right|.$$

In effect, this means that mutational impacts are defined as quantifiable differences between two discrete MD states defined by two stable MD production runs rather than as a property of nonequilibrium dynamics observed during the perturbation of a single MD run by a mutation event. We intentionally avoided basing our conclusions on nonequilibrium dynamics because of recent criticism of modern force fields. It is generally accepted that modern force fields are very accurate in predicting structural and dynamic features of molecular systems (Pérez et al. 2012; Foloppe et al. 2013), particularly when the dynamic states are positioned within the wells of the potential energy surface. However, when system behavior ranges outside of these wells, force field accuracy is currently a matter of debate (Cramer 2004). This method also made for a much more efficient use of computer runtime, as each codon transition state did not require unique MD runs.

### Comparisons of Overall Synonymous and Nonsynonymous Impacts on Molecular Dynamics

Atomic correlations and fluctuations were collected using cpptraj software applied with a 5mer (5 nucleotide) physical mask centered on the codon and filtering only trajectories of the backbone carbon atoms. For all single-based substitutions within the total 64 × 64 codon transition matrix, a list of differences in average correlation and fluctuation (dCORR and dFLUX) calculated over the physical mask for each codon type was derived for both the RNA and DNA contexts. Using the degenerate organization of the real genetic code, overall synonymous and nonsynonymous mutational impacts were taken as the sum of the absolute dFLUX and dCORR over all transition types split into each category (i.e., syn vs. nonsyn). Alternate coding schemes were derived by repeatedly reorganizing an associative array combining all 20 amino acids with all possible letter-base matrix combinations within a 4 × 4 × 4 cube until all (4! x 4! x 4! − 1) = 13,823 alternate coding combinations were attained. The degree of degeneracy for each amino acid was maintained for each of these alternative codes. Synonymous and nonsynonymous mutational impacts were also derived for each alternative code. To assess how the codon organization of the canonical genetic code nonrandomly affected the MD of RNA and DNA polymers, the sum total values of synonymous and nonsynonymous dCORR and dFLUX for the real genetic code were compared to histograms representing all possible alternative codes and subsequently, empirical *p* values were calculated for the synonymous and nonsynonymous contributions using a function for the empirical cumulative distribution programmed in the R statistical programming language.

### Empirical Sequence-Based Comparisons of Overall Synonymous and Nonsynonymous Impacts on DNA Flexibility

For all single-based substitutions within the 64 × 64 codon transitions described above, a list of differences in average DNA flexibility (i.e., dTRX) were also calculated for each codon type. For calculating DNA flexibility, we used the dinucleotide-based TRX score proposed by (Heddi et al. 2010). TRX flexibility scores for whole sequences are simply the sums of the dinucleotide scores divided by the total number of phosphate linkages in the sequence (Eq. 3).

3 $${\text{TR}}{{\text{X}}_{{\text{seq}}}}=\mathop \sum \limits_{{i=1}}^{L} {\text{M}}{{\text{N}}_{{\text{seq}}}}{\text{/}}(L - 1),$$

where *L* is the length of a given sequence, and MN are the respective TRX values for the MN dinucleotides defining the phosphate linkages along this length [Table 1 of (Heddi et al. 2010)]. These dinucleotide values represent the percentage of time that the phosphate linkage between the nucleobases M and N spends in the BII conformation and ranges from 0 = stiff (i.e., entirely BI conformation) to 43 = very flexible (i.e., nearly half time in each conformation). Mutational impacts on DNA (Eq. 4) were defined as the shift occurring in these respective scores when comparing the sequence states before and after a hypothetical mutation event within a codon (i.e., codon transition).

**Table 1:** Optimization of the genetic code to solvated aRNA structures

Method	Mutation	dCORR	dFLUX
		emp. *p* value	emp. *p* value
Implicit	N	0.932	0.994
	S	0.067	0.006
	NN	0.952	0.994
	SN	0.048	0.002
	SS	0.549	0.653
Explicit	N	0.661	0.645
	S	0.338	0.355
	NN	0.937	0.755
	SN	0.010	0.192
	SS	0.987	0.947

Table shows the empirical *p* value (i.e., percent optimization) for the genetic code’s biophysically defined mutational impacts in both implicitly and explicitly solvated simulations when compared to all possible codon reassignments with similar levels of degeneracy

4 $${\text{dTRX}}=\mathop \sum \limits_{{i=1}}^{{N~or~S}} \left| {{\text{TR}}{{\text{X}}^{{\text{before}}}} - {\text{TR}}{{\text{X}}^{{\text{after}}}}} \right|{\text{~/}}i~$$

Using the degenerate organization of the real genetic code, overall synonymous and nonsynonymous mutational impacts were taken as the mean absolute dTRX for each category. Synonymous and nonsynonymous mutational impacts were derived (as in previous section) for each alternative code and similarly used to create distributions of dTRX under random genetic codes to which the dTRX under the standard code could be compared.

## Results

**Fig. 2:**
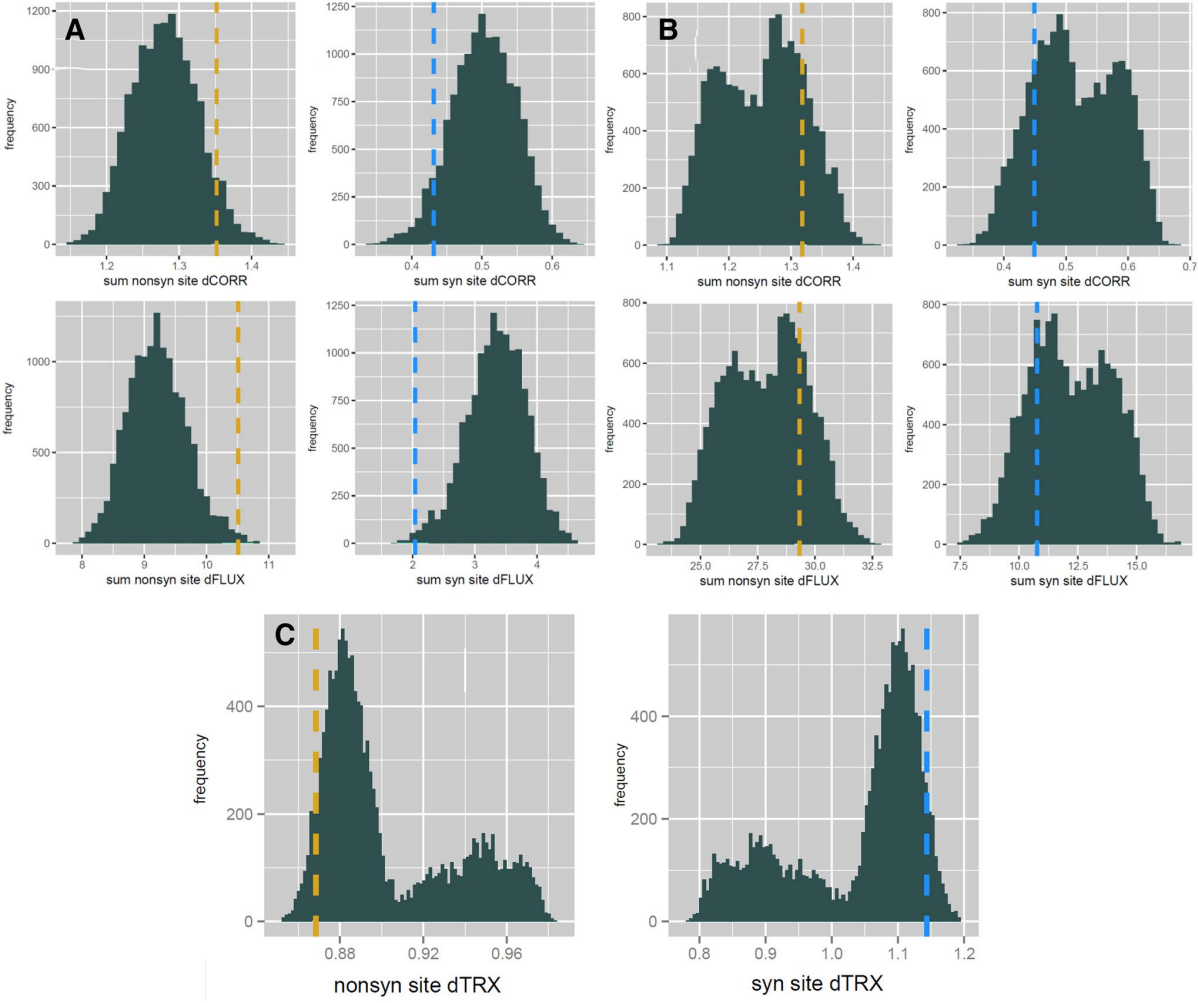
Sum totals of relative impacts of synonymous and nonsynonymous mutation on **a** the MD of double-stranded aRNA, **b** the MD of double-stranded bDNA, and **c** the predicted DNA flexibility when comparing the canonical genetic code (dashed line) to all possible 13,823 alternative codes (histogram). Mutational impacts are defined according to mutational shifts in both correlation of atomic vector trajectories (dCORR—top) and atomic fluctuations (dFLUX—bottom) collected on all backbone carbons within a 5 bp mask centered on the mutation site. The calculations were conducted with cpptraj software (Ambertools16) in plots (**a)** and (**b)** and the TRX score of (Heddi et al. 2010) in plot (**c)**

**Fig. 3:**
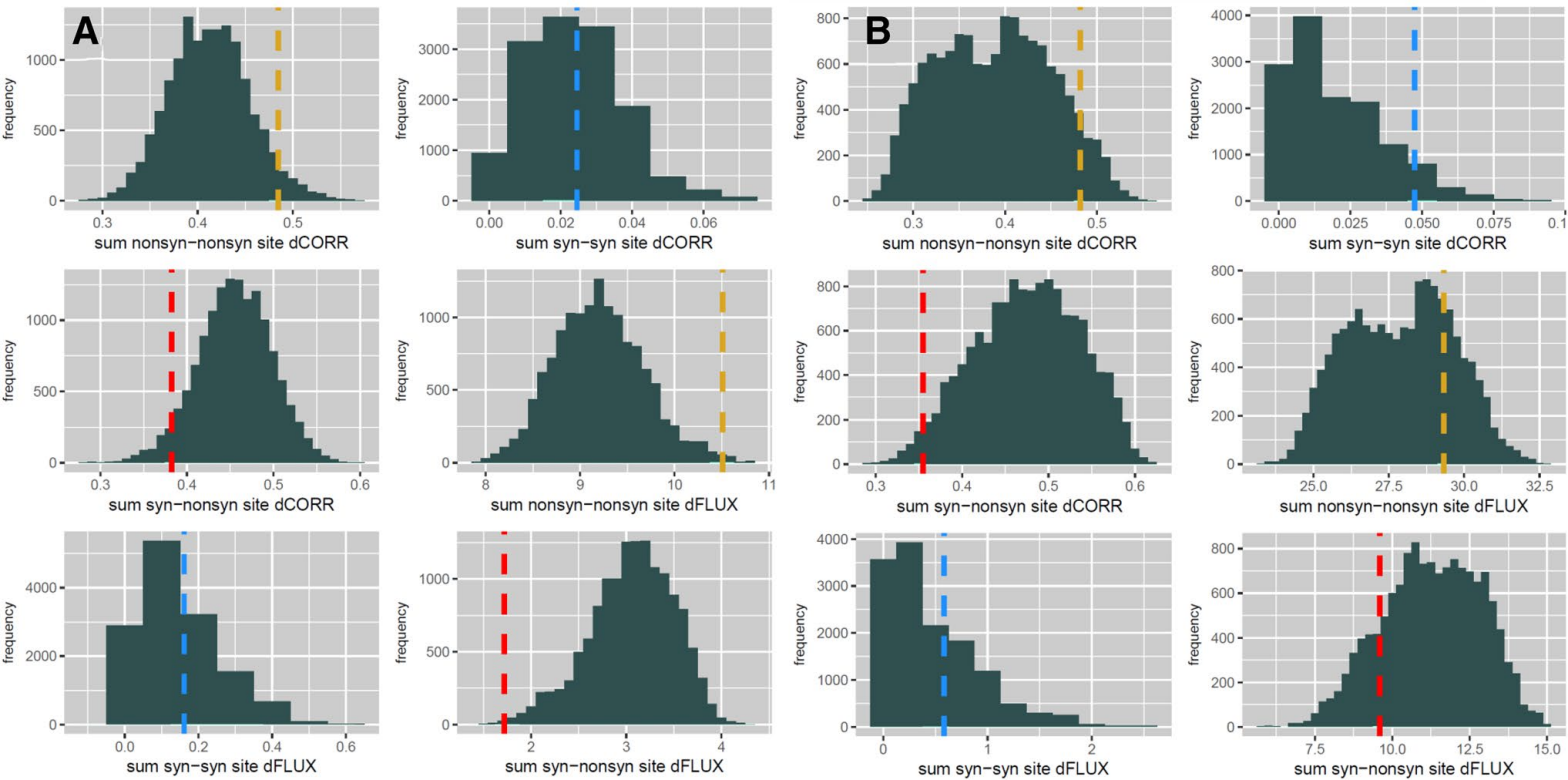
Sum totals of relative impacts of strand-specific mutation on the MD of **a** double-stranded aRNA and **b** bDNA comparing the canonical genetic code (dashed line) to all possible 13,823 alternative codes (histogram). Mutational impacts are defined as in Fig. 2 but were collected according to whether mutation types were symmetric (e.g., nonsynonymous on both leading and lagging strand) or asymmetric (i.e., synonymous on one strand but nonsynonymous on the other)

**Fig. 4:**
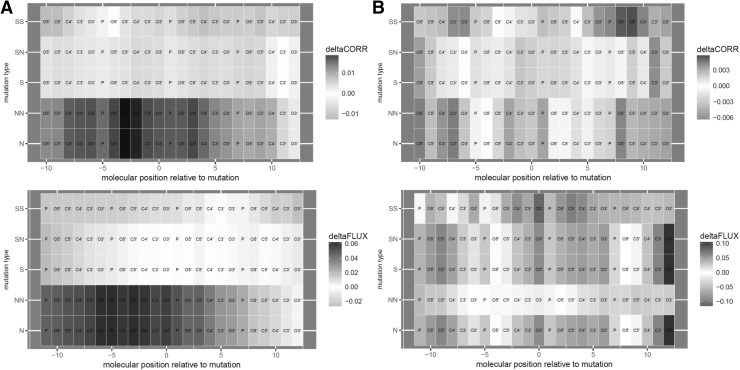
Atom specific heatmaps showing the average dCORR and dFLUX on **a** double-stranded aRNA and **b** bDNA as a function of mutation type (*N* nonsynonymous, *S* synonymous, *NN* nonsynonymous on both strands, *SS* synonymous on both strands, *SN* synonymous on only one strand)

We report that in the context of double-stranded aRNA and to a lesser extent bDNA, the genetic code minimizes the total MD impacts of single-base synonymous mutations (Fig. 2a, b). We also report that the genetic code simultaneously maximizes the mutational impact on DNA flexibility (Fig. 2c). This trend is generally far more pronounced in RNA and is qualitatively consistent regardless of whether implicit or explicit solvation was performed or whether mutational impacts were defined using absolute differences in atomic fluctuation or atomic correlation along the carbon backbone (Tables 1, 2). In solvated RNA structures, the genetic code outperforms (93%/99%) of the 13,823 alternative codes in minimizing the MD impacts (dCORR/dFLUX) of synonymous mutations (Fig. 2a). In the context of DNA, we observe that the genetic code outperforms only (81%/77%) of all alternative codes minimizing (dCORR/dFLUX) of synonymous mutations (Fig. 2b). We compared results based on our MD simulations by performing an identical analysis using a purely sequence-based empirically derived metrics of nucleic acid biophysics, the twist-roll-x displacement (TRX) score for DNA flexibility. Here, we find some similar optimization patterns as well, whereby the genetic code outperforms 88% of all alternatives in maximizing impacts on DNA flexibility (dTRX) of synonymous mutations (Fig. 2c; Table 3). When considering the strand asymmetry of a given mutation [e.g., if it is nonsynonymous or synonymous on both leading and lagging strands when reading in a 5′–3′ direction (i.e., N–N or S–S) or whether it is synonymous on one side but not the other (i.e., S–N)], we find that the trend of minimized impacts of synonymous mutation is much greater when the context is asymmetrical (Fig. 3; Tables 1, 2). Heatmaps of the average positional effects of mutation (dCORR and dFLUX) also reflect obvious differences in synonymous and nonsynonymous mutations in both an RNA and a correlated DNA context, and also demonstrate that the positional effects of mutations are less complex (in patterning) within the context of RNA (Fig. 4). When comparing the correlations among mutational impacts defined by our MD metrics (i.e., dCORR and dFLUX), we find a highly significant correlation between dCORR values for DNA and RNA (*r* = 0.58, *p* < 0.0001) and between dCORR values in DNA and dFLUX values in RNA (*r* = 0.55, *p* < 0.0001). In Supplemental Files A–D, we plot all the RMSD matrices for the equilibration steps of each of the 64 codons in DNA and RNA context. All RMSD plots show evidence of stability prior to each production phase of each MD run.

**Table 2:** Optimization of the genetic code to solvated bDNA structures

Method	Mutation	dCORR	dFLUX
		emp. *p* value	emp. *p* value
Implicit	N	0.809	0.765
	S	0.191	0.235
	NN	0.928	0.785
	SN	0.025	0.163
	SS	0.921	0.674
Explicit	N	0.884	0.992
	S	0.116	0.008
	NN	0.923	0.992
	SN	0.609	0.017
	SS	0.693	0.463

Table shows the empirical *p* value (i.e., percent optimization) for the genetic code’s biophysically defined mutational impacts in both implicitly and explicitly solvated simulations when compared to all possible codon reassignments with similar levels of degeneracy

**Table 3:** Optimization of the genetic code to sequence-based scores for bDNA flexibility (TRX score)

Method	Mutation	emp. *p* value
dTRX	N	0.188
	S	0.882
	NN	0.118
	SN	0.832
	SS	0.851

Table shows the empirical *p* value (i.e., percent optimization) for the genetic code’s biophysically defined mutational impacts for dTRX when compared to all possible codon reassignments with similar levels of degeneracy

## Discussion

We report moderate to very strong patterns of potential functional optimization of nucleic acid MD within the standard codon organization of the genetic code. These impacts are defined via general atomic fluctuations and correlations in atomic vector trajectories on the carbon backbone (i.e., MD simulation), as well as movements that are specific to defining DNA flexibility [i.e., combined twist, roll, and x-displacement of adjacent base pairs predicted by the TRX score of (Heddi et al. 2010)]. The patterns demonstrate an overall minimization of mutational impacts on nucleic acid MD by synonymous changes that are generally much stronger in the context of double-stranded RNA than in DNA. This especially noticeable in atomic fluctuations on RNA where this trend appears extremely optimized (i.e., synonymous impacts of the standard genetic code are in the bottom 1% of dFLUX caused by all random codon organizations). This general trend of minimization of mutational impact on overall backbone dynamics of synonymous sites is accompanied by optimization that maximizes particular movements that facilitate DNA flexibility (i.e., here the synonymous impacts of the standard genetic code are in the top 12% of dTRX caused by all random codon organizations). Therefore, it appears that while mutational impacts of synonymous sites are most organized so as to minimize mutational impacts that affect random thermal motion in RNA, they also serve to simultaneously facilitate the combinations of adjacent base pair motions that serve to maximize polymer flexibility in DNA. Because a sequence-based predictor of double-stranded RNA flexibility does not exist, we could not test whether the facilitation of RNA flexibility was also unusually accommodated by the genetic code. However, the strong minimization of synonymous impacts we observe in simulation regarding RNA backbone fluctuation is suggestive that the phenomena of dual-use codons and other forms of genetic code optimization observed in the context of chromatin-bound eukaryotic DNA, may extend from preadaptation of the RNA world to stabilize thermal characteristics of RNA duplexes in the face of mutation.

Some mutations are asymmetric in their effect on amino acid replacement under a given codon table (i.e., classified here as ‘syn–nonsyn’ or synonymous in the reading frame on one backbone while nonsynonymous on the other). A much smaller category of synonymous mutations is symmetric (i.e., ‘syn–syn’). We find that these asymmetric synonymous changes are highly optimized under the standard code while symmetric synonymous changes clearly are not. We do not have a definitive explanation for this, but might suggest that the optimization of the code regarding MD of duplexed RNA arose within a simpler doublet code whereupon fewer base substitutions were synonymous prior to later expansion to the triplet code we have today.

The nonrandom pattern of optimization in synonymous mutation we observed was quantitatively and qualitatively consistent regardless of the method of solvation within the MD simulation we chose. This result is also highly consistent regardless of whether we defined the impact of mutation through its effect on the carbon backbone via atomic fluctuation (i.e., beta factors representing rapid harmonic bond vibrations) or atomic correlations (i.e., representing similarities and differences in the vector trajectories of adjacent atoms). The optimization of we observe using an empirically based sequence-dependent metric of DNA flexibility (TRX score) corroborates our earlier findings that changes to DNA flexibility are mitigated by synonymous mutation affecting GC3 in coding regions (Babbitt and Schulze 2012; Babbitt et al. 2014), and also further indicates that our main results are not likely an artefactual result of the process of MD simulation. In short, both our computer-intensive MD modeling and simple sequence-based scoring converge to tell a similar story that the standard genetic code is likely highly optimized to several particular biophysical aspects of nucleic acids.

### Is the Triplet-Based Genetic Code a Functionally Accessorization of an Earlier Doublet Code?

As nonsynonymous sites are defined by their potential effects on protein function, we argue that this optimization of synonymous mutation within the standard codon organization supports the existence of an adaptively evolved functional role for synonymous sites in facilitating changes in nucleic acid polymer dynamics independently of protein evolution. In accordance with recent studies highlighting both the torsion-based mechanics that may underlie transcriptional regulation, as well as the detection of strong purifying selection on synonymous sites, we argue that the triplet codon may very likely represent an ancient evolutionary event predating LUCA in which a simpler doublet code was expanded into triplets through a form of ‘functional accessorization.’ The functional adaptation of triplets enabled more diverse and complex physical interactions between protein-encoding nucleic acid polymers that would not directly limit or interfere with amino acid specification. In an early RNA–protein world, this functional aspect of synonymous sites may have been restricted mainly to RNA duplexing interactions at a time before the strict singular roles of storage versus messenger had evolved in RNA. The fundamental correlations between MD of DNA and RNA, especially pronounced within unbalanced GC backgrounds, may indicate that much of what we observe in RNA has preadapted DNA to be similarly optimized. Therefore, the functional adaptation of synonymous sites in RNA could have later been incorporated into prokaryotic DNA-based systems and eukaryotic chromatin-based systems as well. Such a scenario, if true, would potentially unify the three current theories of the origin of the genetic code, unnecessarily categorized under physicochemical, adaptive and co-evolutionary hypotheses. In our scenario, each of these basic ideas are intertwined. Additionally, our hypothesis that the genetic code underwent a second period of adaptive expansion in response to an entirely different selection pressure than what molded earlier adaptive events that minimized mutational impacts on a ‘proto-ribosome’ would make it naturally difficult to unify the potential evolution of the code under one singular idea, as Crick, and many others had earlier failed to do (Cobb 2015). We would also add that Crick’s ‘frozen accident’ hypothesis is also a rather implausible result under the modern view of how functional conservation works at a molecular level. If the codon organization was critical to molecular function, its evolutionary and organizational patterns would not likely be ‘frozen’ in a random or ‘accidental’ state of affairs, as selection always tends to preserve nonrandom ‘non-accidental’ features of molecular organization at all other levels of sequence evolution.

## Conclusion

The fact that we refer to the systematic way that templated polymerization occurs in the cell as a ‘code’ belies a deeply embedded linguistic metaphor in the biological sciences (Cobb 2013, 2015). Outside of this context, codes and ciphers generally imply messages that are intended to be hidden in abstraction (often during wartime). However, there is no evidence that the codon organization of the genetic code ever shared this property of human-invented codes. In fact, the near universality of the genetic code greatly enables viral pathogenicity and implies that if the code was once held secret from potential parasites of LUCA, that secret has long since been compromised. A less common, but perhaps more accurate metaphor is that the genetic code represents a ‘language’ of life (Cobb 2015). This implies something rather different, as unlike the fully abstract symbolic representation that typically embodies a code, a spoken or written language, like all other forms of signal communication, are only partly abstracted from the physical properties of the media through which communication occurs. Many examples of this principle exist in linguistics (Barney et al. 2012; Kumar et al. 2016; Hickok 2016), graphemics (Jean and Oates 1992), electronic communications engineering (Gertner 2013), and even communication through animal behavior (Endler et al. 2005; Clark and Feo 2010; Burtt et al. 2011). Remarkably, despite the pervasiveness of this linguistic metaphor in the central dogma of molecular biology, where ‘information’ regarding protein is ‘transmitted’ via the media of solvated nucleic acids (Cobb 2013), we have never directly investigated the potential adaptation of the genetic code to the MD characteristics of the media that carry this information (i.e., solvated DNA and RNA helix structures). Our work here is an initial step to remedy this gap in our understanding of the ‘code.’

We conclude that the recently proposed ‘dual use’ nature of the triplet codon (Stergachis et al. 2013) was preadapted in the MD context of double-stranded RNA. We hypothesize that the dual-use triplet codon was the result of an adaptive expansion of a primordial doublet-based genetic code, perhaps already adapted to minimize impacts on RNA stability and translational error (Koonin and Novozhilov 2009), and is functional in its ability to facilitate specific MD movements necessary for more finely tuned transcriptional control governed by polymer flexibility that can enhance nucleic acid self-interaction and/or interaction with binding proteins. This ancient expansion event over 3.5 billion years ago may have served to functionally accessorize a primordial doublet-based genetic code, so as to multiplex gene regulatory information rooted in nucleic acid biophysics without interference with amino-acid specification in proteins.

## Electronic supplementary material

Below is the link to the electronic supplementary material.

Supplementary material 1—Supplemental Files A–D. Root mean square deviation (RMSD) heatmaps for all 64 equilibration MD runs in (A) implicitly solvated DNA, (B) implicitly solvated aRNA, (C) explicitly solvated DNA, (D) explicitly solvated aRNA. (PDF 10193 KB)

Supplementary material 2 (PDF 10308 KB)

Supplementary material 3 (PDF 8801 KB)

Supplementary material 4 (PDF 8100 KB)
